# Exploring Various Transfection Approaches and Their Applications in Studying the Regenerative Potential of Dental Pulp Stem Cells

**DOI:** 10.3390/cimb45120626

**Published:** 2023-12-13

**Authors:** Hanaa Alkharobi

**Affiliations:** Department of Oral Biology, College of Dentistry, King Abdul-Aziz University, Jeddah 21589, Saudi Arabia; halkharobi@kau.edu.sa; Tel.: +966-553353700; Fax: +966-126951996

**Keywords:** transfection, dental pulp, stem cell, regeneration, dental stem cell, regenerative medicine

## Abstract

Transfection is a contemporary approach for introducing foreign genetic material into target cells. The effective transport of genetic materials into cells is mostly influenced by (a) the characteristics of the genetic material (quantity and quality), (b) the transfection procedure (incubation time, ratio of the reagents to the introduced genetic material, and components of cell culture), and (c) targeted cells for transfection (cell origin and cell type). This review summarizes the findings of different studies focusing on various transfection approaches and their applications to explore the regenerative potential of dental pulp stem cells (DPSCs). Several databases, including Scopus, Google Scholar, and PubMed, were searched to obtain the literature for the current review. Different keywords were used as key terms in the search. Approximately 200 articles were retained after removing duplicates from different databases. Articles published in English that discussed different transfection approaches were included. Several sources were excluded because they did not meet the inclusion criteria. Approximately 70 relevant published sources were included in the final stage to achieve the study objectives. This review demonstrated that no single transfection system is applicable to all cases and the various cell types with no side effects. Further studies are needed to focus on optimizing process parameters, decreasing the toxicity and side effects of available transfection techniques, and increasing their efficiencies. Moreover, this review sheds light on the impact of using different valuable transfection approaches to investigate the regenerative potential of DPSCs.

## 1. Introduction

Transfection is a method by which genomic materials (transgenes) of interest are transferred into target cells to study and modulate specific cellular functions [1,2,3,4] and understand the molecular mechanisms and pathways underlying different cellular processes. This is crucial for identifying specific biomarkers that play various roles in disease diagnosis, prognosis, and treatment [5,6]. Transfection is also used in gene therapy to treat untreatable inherited genetic disorders.

The first gene delivery system was used in 1989 in tumor infiltrating lymphocytes [7]. The first gene therapy was performed in 1990 using the adenosine deaminase (ADA) gene to treat patients with severe combined immunodeficiency [8]. Interestingly, the development of existing technologies has allowed researchers to deliver different types of nucleic acids, such as DNA, RNA, and non-coding RNA, including small interfering RNA (siRNA), short inhibitory RNA (shRNA), and microRNA (miRNA), into target cells [9,10,11,12]. miRNA is a single-stranded non-coding small RNA composed of approximately 22 nucleotides. It plays a pivotal regulatory role in several cellular activities, such as proliferation, differentiation, and apoptosis. A review of the literature revealed that different miRNAs play important regulatory roles in the differentiation capacity of stem cells. Several studies have investigated the role of various miRNAs, including miR-145, miR-143-3p, miR-140-5p, miR-488, miR218, miR-125a-3p, miR-27a-5p, miR-223, miR-21, miR-143, miR-215, miR-219a-1-3p, miR-31, miR496, miR-218, miR-24-3p, miR-146a-5p, miR-196a, miR-188-3p, miR-424, miR-378a, miR-135, and miR124, in the modulation of cellular activities [13]. 

**Figure 1:**
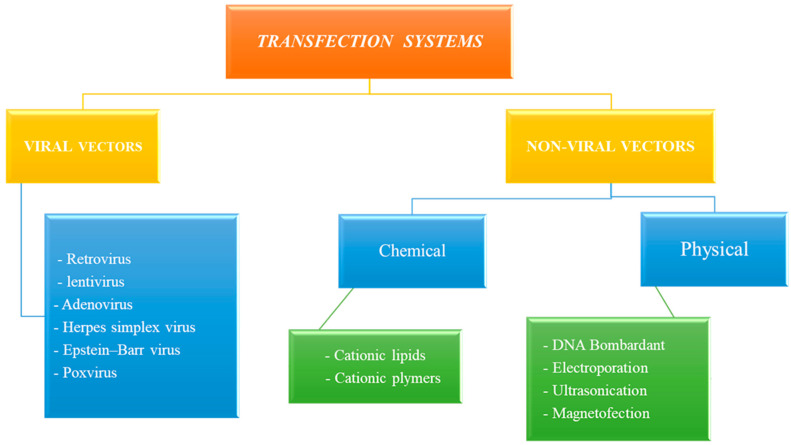
Multiple approaches of transfection systems.

Transfection can be performed using (1) viral-based systems (e.g., retrovirus, adenovirus, herpes simplex virus, pox virus, lentivirus, and Epstein–Barr virus) and (2) non-viral-based systems, including physical approaches (e.g., naked DNA, DNA bombardment, electroporation, hydrodynamics, ultrasound, and magnetofection) and chemical approaches (e.g., cationic lipids, cationic polymers, and lipid polymers) (Figure 1).

Dental pulp is mainly composed of neural fibers, blood, and lymphatic vessels. In addition, it contains mesenchymal stem cells known as dental pulp stem cells (DPSCs). DPSCs perform several functions, including the maintenance of tooth viability, tissue protection, and tissue homeostasis [14]. DPSCs are a promising and easy-to-harvest source of cells that express mesenchymal stem cell markers with good proliferation and differentiation abilities. However, these cells are usually discarded as biological waste in dental clinics [15]. Recent studies have revealed the distinct differentiation potential of DPSCs into different lineages, including odontogenic, osteogenic, chondrogenic, neurogenic, and angiogenic [16]. In addition, DPSCs are a good source for cell-homing because they are found in the root canal and have the ability to regenerate [17]. Studies have shown similarities between DPSCs and bone marrow stromal cells, which improve the application of DPSCs in musculoskeletal regenerative medicine [15]. These cells are also targeted in research as a source of autologous cells, which are of great interest in the field of regenerative medicine, both inside and outside the oral cavity [18]. Table 1 summarizes the key properties of DPSCs. DPSCs have demonstrated their ability to promote proper migration and angiogenesis and are considered a good source of cells to regenerate gingival, dental pulp, bone, and periodontal tissue. However, DPSCs show higher proliferation rates than stem cells from adipose tissue, which in turn demonstrates higher clonogenicity [14]. Genova et al. demonstrated that DPSCs (isolated from the third molar) and buccal fat pad stem cells (BFP-SCs) are promising sources of mesenchymal stem cells that can be used for regenerative treatments. BFP-SCs and DPSCs can differentiate into osteoblast-like cells and produce a mineralized matrix [19]. Moreover, stem cells have been isolated and characterized from other dental tissues, including stem cells from human exfoliated deciduous teeth, periodontal ligament stem cells, stem cells from apical papilla, and dental follicle progenitor cells. These cells express a self-renewal ability and multilineage differentiation potential, including into odontogenic, osteogenic, chondrogenic, adipogenic, myogenic, and neurogenic cells [20].

Studying and modulating specific molecules in DPSCs using different optimized transfection approaches could have a significant impact on cell function, including proliferation and differentiation potentials. The insertion of genes or molecules into DPSCs can help control the differentiation (osteogenic/chondrogenic/angiogenic) potential of these cells, prevent their apoptosis, and enhance their proliferation capacity. DPSCs have also been transfected to enhance their survival rate and encode various therapeutic proteins, subsequently improving their therapeutic potential.

Therefore, the current literature review aims to examine various transfection approaches (cons and pros), particularly the applications of these transfection techniques in investigating the regenerative potential of DPSCs. 

## 2. Methodology

**Figure 2:**
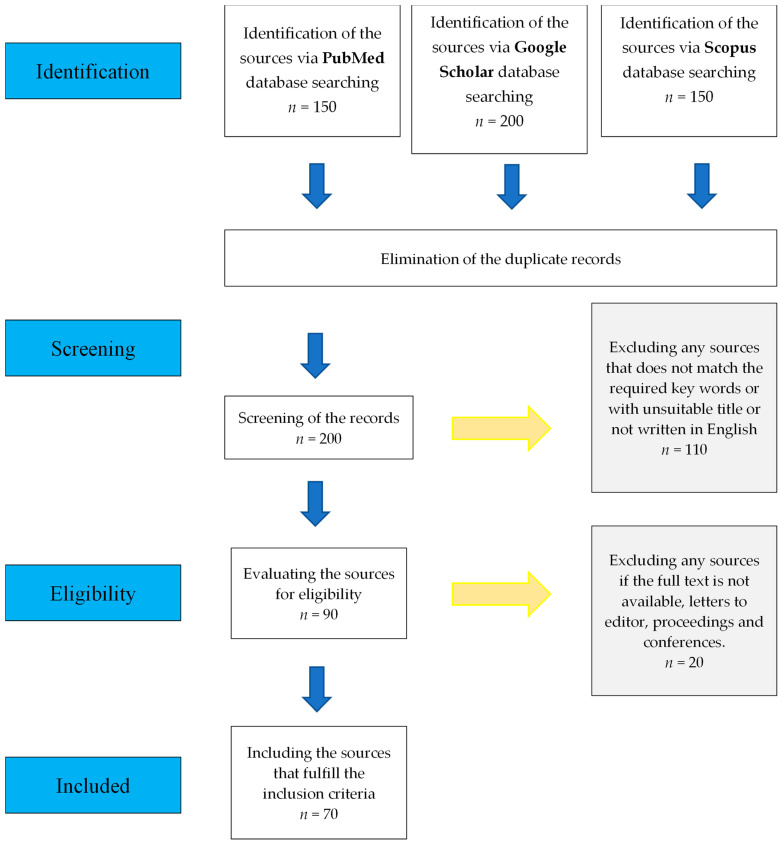
The workflow of literature selection.

Several databases, including Scopus, Google Scholar, and PubMed, were searched to obtain the literature for the current review. The keywords used during the search included “transfection”, “dental stem cells”, “dental pulp stem cells”, “dental stem cell”, “regenerative medicine”, “regeneration”, and other related key terms. The primary search returned approximately 500 articles and published protocols from various databases that discussed different transfection methods, efficiency assessment methods, and transfection applications, particularly in exploring the regenerative potential of DPSCs. Approximately 200 articles were retained after removing duplicates obtained from different databases. Articles published in English that discussed different transfection approaches were included. Several sources were excluded because they did not meet the inclusion criteria. Approximately 70 relevant published sources were included in the final stage to achieve the objectives of this study (Figure 2).

## 3. Transfection Approaches

### 3.1. Viral Approach

Viral approaches involve the use of viral vectors to deliver nucleic acids (DNAs or RNAs) into target cells [27]. In this approach, viral vectors are modified by deleting some areas in their genomes such that their replication becomes defective, making them safe to use [28]. Although viral transfection systems allow practical and stable expression of transgenes within target cells, their application is limited by several disadvantages, including risky preparation and restricted transgene size [29,30]. The immune response is also problematic in viral transfection systems because the interaction of the virus with the host antibodies triggers inflammation and toxin production, which may cause the degeneration of surrounding tissues [28]. 

Retroviruses can pass through the nuclear pores of mitotic cells. Therefore, they can be used to transfect dividing cells [28,31]. However, they cannot transduce non-dividing cells and thus have low efficiency in vivo [31]. In contrast, lentiviruses (a subclass of retroviruses) can be integrated into non-dividing cells with fewer adverse side-effects and are extensively used for gene transfer in the central nervous system. Adenoviruses can deliver large DNA particles (up to 38 kb) [32]; however, the delivered gene is only transiently expressed because the transferred gene cannot integrate into the host genome. Interestingly, non-enveloped adeno-associated virus (AAV) is a promising and safe viral vector that has been used in clinical experimental research for therapeutic purposes. A previous study produced recombinant AAV particles containing targeted therapeutic DNA sequences without eliciting the expression of viral genes [30], thereby reducing the potential immunogenicity of the viral vector. Given their neurotropic features, herpes vectors possess excellent potential for delivering genes to tumors and cancer cells in the nervous system [33]. The Epstein–Barr virus can transfer large DNA fragments into target cells, and it is appropriate for long-term effects. Poxviruses are stable vectors that allow homologous recombination and in vitro ligation [28]. These studies have demonstrated the frequent use of the viral approach for various purposes, such as vaccines and cancer schemes [34]. Table 2 shows a descriptive comparison of viral vectors.

### 3.2. Non-Viral Approach

Recently, non-viral delivery systems have been developed as safer and cheaper alternatives to viral delivery systems. Although non-viral systems are less efficient for gene delivery than viral systems, they demonstrate lower immunogenicity. Moreover, unlike viral systems, non-viral systems are not restricted by the size of the transgenic DNA [35]. Non-viral systems can be implemented either as naked DNA or through physical (particle bombardment using a gene gun, electroporation, ultrasound utilization, and magnetofection) or chemical (cationic and polymers) facilitator methods.

#### 3.2.1. Naked DNA Approach

Naked DNA is DNA that is not associated with protective molecules (proteins or lipids). Naked DNA injection, without any carrier, is possibly one of the most straightforward and nontoxic approaches for gene delivery. Gene transfer using a naked DNA approach is known as transformation, and it is generally considered a laboratory procedure. The delivery of genetic materials (e.g., DNA [2–19 kb]) can cause long-term expression (more than 19 months) of the transfected gene in various cells (skeletal or cardiac muscle, skin, thymus, and liver) when injected directly [36,37]. 

Naked DNA injection is a straightforward and harmless method; however, it shows low efficiency for gene transfection and has limited applications. It can be used several times without inducing a serious immune response. This can aid in the use of DNA vaccines against different diseases, such as AIDS and tumors. The mechanism that mediates naked DNA–gene transfer can be enhanced using different methods, including external physical forces and a wide range of chemicals.

#### 3.2.2. Physical Approach

Physical approaches involve the use of mechanical, electrical, ultrasonic, and magnetic forces to facilitate rapid and transient penetration of genetic materials across the outer membrane of the target cell. 

Genetic particle bombardment is achieved by coating plasmid DNA with gold or tungsten spherical particles (1–3 µm in diameter), whose penetration into target cells is then accelerated by pressurized gas. This approach is presently used to deliver genes into mammalian cells, especially for DNA-based immunization or vaccination [37,38].

Electroporation involves the use of intermittent high-voltage electrical pulses to make target cells temporarily permeable and to dispense genetic material into their nuclei [39]. Electroporation parameters, including the voltage, pulsation mode, and pulse number, must be optimized to achieve the best transfection efficiency. For example, the optimal electroporation parameters to transfect DPSCs are 100 V, 20 ms, and one-pulse square-wave condition with a 1:1 DNA (µg): Lipofectamine 2000 (µL) ratio [39]. The size and concentration of the plasmid DNA also influence the efficacy of the delivery system [40,41]. For example, ultrasonication creates nanometric pores in the membrane to facilitate the intracellular delivery of small genetic particles into target cells; however, its application is limited by its low efficiency.

Magnetofection is a delivery system that utilizes magnetic particles to generate high-magnetic field pulses that permeabilize cell membranes and concentrate nucleic-acid-containing particles into target cells. However, the exact mechanisms underlying this approach remain poorly understood. Interestingly, this delivery system is customizable for different types of nucleic acids and compatible with different transfection reagents. Furthermore, it has been successfully tested in various cell lines, including hard-to-transfect and primary cells [42]. Magnetofection kits are commercially available and contain spherical magnetic nanoparticles that are positively charged to facilitate the formation of complexes between the magnetic nanoparticles and the target genetic material that needs to be transfected. 

#### 3.2.3. Chemical Approach

Chemical systems are more common than physical methods, and they have achieved satisfactory results in the preclinical and clinical fields. These systems are composed of particles that encapsulate and protect genetic materials from external DNase until they penetrate the target cells and effectively release the loaded DNA in a low-acidic environment (pH 3.0). However, chemical systems face challenges that need to be addressed [43]. 

Cationic gene delivery systems are advantageous over virus-based systems owing to their improved safety, lower toxicity, lower immunogenicity, and capacity to carry genetic materials of different sizes. However, they tend to show lower gene expression levels than viral systems. 

Cationic systems deliver genetic materials via non-specific interactions between cationic particles and the cell surface, endocytosis into vesicles, compaction, release of DNA particles from endosomes, and delivery of particles to the nucleus. Cationic lipids and polymers are the commonly used cationic particles. Cationic lipid-based systems (cationic liposomes) are composed only of biological lipids, including lipoplexes and stabilized plasmid–lipid particles (SPLPs) [29,44]. However, these systems demonstrate rapid liposome degradation, which decreases the persistent expression of the transfected genes. This phenomenon can be improved by modifying the liposomal surface using hydrophilic polymers [45].

Mortimer et al. [29] controlled the cell cycle of target cells to enhance cationic lipid-mediated transfection. They found that arresting target cells in the G1 phase in the presence of lipoplexes or SPLPs enhanced the stability of transgene expression. Moreover, incubating the cells with lipoplexes or SPLPs during or before mitosis increased the expression of reporter genes [29].

New generations of liposomes, such as stealth, targeted, and stimuli-sensitive liposomes, have been introduced to improve the performance of conventional liposomes. Lipid–polymer systems with an advanced three-part system have also been developed. In this system, DNA is first precondensed with polycations and then coated with either liposomes or amphiphilic polymers with or without helper lipids [28]. The efficiency of cationic liposome delivery systems depends on the amount, size, and structure of the liposomes. The charge of cationic polymers also influences their ability to bind to the transferred genetic material. For instance, cationic polymers with high molecular weights and net positive charges have high binding ability and stability. As the length of a polymer increases, its transfection ability and toxicity increase [46].

## 4. Transfection Outcomes

### 4.1. Stable Transfection

Stable transfection aims for long-term stable expression of the genetic material delivered into target cells. This method is performed either through the integration of foreign DNA and the host nuclear genome or the use of an episomal vector preserved in the host nucleus, which leads to transgene expression even after replication of the transfected cells. Therefore, stable transfection is preferred in long-term genetic and pharmacological studies, such as when protein production is required [12]. 

### 4.2. Transient Transfection 

Transient transfection is useful for studying short-term expression of transgenes. Cells can be harvested 24–96 h post transfection and used for RNA interference-mediated gene silencing or the rapid production of recombinant proteins on a small scale. Transient transfection using mRNA produces rapid results (within minutes) because mRNA is expressed in the cytoplasm without the need to be transcribed in the nucleus. In transient transfection, nucleic acids are delivered as plasmids or oligonucleotides into target cells, leading to the loss of transgene expression during cell replication [12].

## 5. Uses of Transfection in Studying and Enhancing the Regenerative Capacity of DPSCs

DPSCs are mesenchymal stem cells in the cell-rich zone of the dental pulp. Normal biological processes in DPSCs are potentially affected by disease or inflammation, which influence the ability of these cells to differentiate or regenerate efficiently [47]. DPSCs possess mesenchymal stem cell characteristics, including self-renewal, multilineage differentiation, and immunomodulation, with substantial potential for regenerative therapy, making them targets for research and future clinical applications in regenerative medicine [39,48]. Currently available transfection approaches allow us to study the effects of the differential expression of various genes related to different cellular processes, such as proliferation, cell cycle, aging, apoptosis, and the regeneration potential of DPSCs [49]. 

### 5.1. Studying the Odontogenic Potential of DPSCs

siRNAs have been efficiently transfected into DPSCs using Lipofectamine RNA iMAX (Life Technologies, Carlsbad, CA, USA)-siRNA duplex under specific culture conditions to study the effect of knocking down or silencing the target genes on the odontogenic ability of DPSCs. A previous study showed that silencing growth/differentiation factor 11 (*GDF11*) reduces the odontogenic differentiation potential of DPSCs, indicating that *GDF11* is crucial for the odontogenic differentiation of these cells. This finding was confirmed by decreased alkaline phosphatase (ALP) activity and Alizarin Red staining, in addition to the downregulation of odontogenic-related gene expression [50]. 

An electroporation-mediated gene delivery approach was used to transfect DPSCs with the *GDF11* gene. The overexpression of recombinant human *GDF11* in DPSCs stimulated the expression of dentin sialoprotein, a differentiation marker for odontoblasts. This suggests that *GDF11* is a potential factor for healing injured pulp tissues, and it can be used for endodontic treatment [51]. The study also utilized another transfection approach, sonoporation, which involved the transfer of *GDF11* plasmid DNA into DPSCs. In this approach, the cells were treated with plasmid pEGFP or CMV-LacZ in 5–10% Optison and then transfected using ultrasound (1 MHz, 0.5 W/cm^2^, 30 s), leading to efficient gene transfer. The study demonstrated that high *GDF11* expression stimulated the expression of differentiation markers for odontoblasts [51]. 

In contrast, studies have reported that the downregulation of Wnt4 expression in DPSCs reduces their odontogenic differentiation potential. The Wnt4-mediated odontogenic differentiation of DPSCs was investigated by transfecting DPSCs with a recombinant Wnt4-siRNA via a lentiviral transfection system [52]. 

In another study, DPSCs were transfected with vascular endothelial growth factor (VEGF) and stromal cell-derived factor-1α (SDF-1α) to investigate the application of human DPSCs in dental pulp regeneration. In that study, lentiviral carriers were used to deliver VEGF and SDF-1α genes into DPSCs, resulting in the overexpression of both genes. Compared with non-transfected DPSCs, the transfected cells showed significantly higher proliferation and endothelial cell migration and developed a vascular tube on Matrigel in vitro [53]. The study also used the transfection technique to examine the role of vascular endothelial growth factor receptor-2 (*VEGFR-2*) in the odontoblast differentiation of DPSCs. These cells were transfected with shRNA using viral vectors to silence *VEGFR-2* gene expression. Silencing of *VEGFR-2* downregulated the expression of dentin matrix proteins, such as dentin matrix protein-1, dentin sialoprotein, and bone sialoprotein. These findings suggest the importance of *VEGFR-2* as a potential modulator of the dentinogenesis capacity of DPSCs [54]. 

Transfection proficiency also identified the role of the ERK signaling pathway in familial non-syndromic oligodontia. The transfection was successfully conducted under standard culture conditions using a newly designed magnetic nanocarrier, GCC-Fe_3_O_4_. The results showed that the nanocarrier could attain serum-endurable properties with approximately 90% transfection efficiency [55]. 

In another study, DPSCs transfected with a mutant MSX1 plasmid showed lower odontogenic potential, as demonstrated by decreased expression levels of dentin sialophosphoprotein (DSPP) and bone sialoprotein. Moreover, the transfection technique used in the study downregulated the expression of other odontogenic genes and affected the formation of mineralized nodules [56].

Liu et al. performed loss-of-function and gain-of-function experiments to induce the differentiation potential of DPSCs and enhance the expression of DSPP and dentin matrix protein 1 (DMP1). This was accomplished by repressing miR-145 and miR-143, as they can bind and inhibit the 3′-UTRs of Krüppel-like factor 4 and osterix genes. Moreover, Yang et al. showed that the downregulation of miR-143-3p leads to the upregulation of nuclear factor-κB (RANK) and the osteoprotegerin (OPG)/nuclear factor-κB ligand (RANKL) signaling pathway and the induction of DPSCs to form odontoblasts. Simultaneously, the downregulation of miR-143-3p inhibited the cycle progression of DPSCs and induced apoptosis through the activation of the OPG/RANKL/RANK pathway [13].

### 5.2. Studying Osteogenic and Chondrogenic Differentiation Potential of DPSCs 

Wnt signaling is vital for the osteogenic differentiation of stem cells. Inflammation affects the osteogenic potential of DPSCs and downregulates Wnt4 [57]. Therefore, DPSCs with inflammation show lower osteogenic differentiation potential than those without inflammation. The delivery of Wnt4 into inflamed stem cells using lentiviral vectors restores Wnt4 expression in these cells and improves their osteogenic differentiation potential [47].

A study demonstrated that the transient transfection of DPSCs with a BMP7 gene plasmid (recombinant pcDNA3.1/V5 His TOPO BMP7) induces their osteogenic differentiation by increasing the expression of key osteogenic factors, such as ALP and osteocalcin [58]. 

A recent study used electroporation to transfect DPSCs with a transforming growth factor-beta 1 (TGF-B1) gene plasmid in the presence of 10% platelet-rich plasma (PRP). They concluded that DPSCs transfected with the TGF-B1 gene and cultured in 10% PRP medium have a great chondrogenic potential. Moreover, continuous TGF-β1 overexpression in DPSCs stimulated osteogenic and chondrogenic differentiation, increased the proliferation rate, and decreased the total apoptosis and cellular senescence of these cells. However, TGF-β1 overexpression did not influence the immunophenotype or expression of stemness-related surface markers [49].

The Ena/VASP-like protein (EVL) plays a pivotal role in many biological processes. A study investigated the underlying mechanism of EVL control of the osteogenic differentiation of DPSCs using siRNAs specifically targeting EVL (si-EVL). The expression of EVL was increased in the DPSCs owing to the EVL gene, which was subcloned into the pEZ-M98 vector to generate a pM-EVL plasmid. The results showed that EVL overexpression considerably enhanced the osteogenic capacity of DPSCs, which was confirmed by ALP staining, ALP activity, mineralized nodule formation, and the expression of osteogenic differentiation genes. Interestingly, EVL demonstrated its ability to activate the JNK pathway, resulting in the phosphorylation of p38 MAPK during DPSC differentiation [59]. 

The transfection technique also helped another group investigate how lysophosphatidic acid (LPA) signaling plays a role in the proliferation and osteogenic differentiation of DPSCs. They transfected DPSCs with siRNAs for human *LPAR3* and non-specific control siRNA: siLPAR3, 5′-GCCUAUGUAUUCCUGAUGUTT-3′ (sense) 5′-ACAUCAGGAAUACAUAGGCTT-3′ (antisense); siControl, 5′-UUCUCCGAACGUGUCACGUTT-3′ (sense), and 5′-ACGUGACACGUUCGGAGAATT-3′ (antisense). After 6 h of incubating DPSCs with the transfection reagents, the transfection medium was replaced with a fresh medium. Proliferation was measured using the cell counting kit-8 assay. Osteogenic differentiation was confirmed using ALP staining, ALP activity measurements, and RT-qPCR. siRNA-mediated *LPAR3* silencing and extracellular signal-regulated (ERK)/mitogen-activated protein (MAP) kinase inhibitors were used to further explore the molecular mechanisms underlying the role of LPA in the proliferation and differentiation of DPSCs [60].

Zhang et al. demonstrated that the overexpression of miR-143 inhibited the osteogenic differentiation ability of DPSCs. In addition, they elucidated that tumor necrosis factor alpha (TNF-α) was a target of miR-143 in DPSCs and that miR-143 can regulate TNF-α expression through post-transcriptional binding to its 3′-UTR, leading to the inhibition of TNFα-induced osteogenic differentiation of DPSCs. This was proven by the inhibition of miR-143, which in turn enhanced the osteogenic differentiation of DPSCs through the activation of the NF-κB signaling pathway [13]. 

### 5.3. Other Applications

Soda et al. demonstrated the advantage of transfection in augmenting the production of induced pluripotent stem cells (iPSCs). Pluripotent stem cells can be induced by transfecting dental pulp cells isolated from deciduous teeth with the transient reprogramming Yamanaka factors. Repetitive transfections (≤3) contribute to improved iPSC generation, thus showing superior multipotentiality within weeks post transfection of these cells compared with untransfected cells of the exact origin [61]. However, this multipotentiality phenotype undergoes a stepwise loss during differentiation. 

A previous study investigated the role of DPSCs in the spinal cord injury repair model under specific conditions. AAV particles were used to transfect DPSCs with basic fibroblast growth factor (bFGF), and a 90% transduction rate was achieved. These cells were cultured under specific hypoxic (<1% O_2_) conditions for at least 6 h. The study suggested that high bFGF expression in DPSCs helps stimulate the recovery of patients with spinal cord injury in a hypoxic environment [62].

Another study transfected DPSCs with miR-124 to assess the potential effect of miR-124 in modification of neural protein marker (Nestin, NOTCH1, and SOX2) and neural marker (β-tubulin III, GATA-3, and peripherin) expression. Quantitative reverse transcription-PCR (qRT-PCR) and immunofluorescence staining were used to study the expression patterns of neural progenitor markers. At the genetic level, Nestin was upregulated 6 h post transfection; however, its expression decreased at 24 and 48 h post transfection [63]. Meanwhile, β-tubulin III showed high levels at 6 and 16 h post transfection and decreased expression at 48 h post transfection. At the protein level, transfection of DPSCs with miR-124 only affected Nestin among the studied neural progenitor and neural markers [63]. 

Researchers are interested in the proliferative ability of DPSCs because of their clinical applications in tissue repair and regeneration. A recent study investigated the role of miR-584 and transcriptional co-activator with PDZ-binding motif (TAZ) in DPSCs. Both miR-584 and TAZ play important roles in various biological processes of DPSCs, including cell proliferation and differentiation. The study transiently transfected miR-584 mimics and miR-584 inhibitors into DPSCs. The study found that upregulated miR-584 expression and downregulated TAZ expression levels were different between aging and young dental pulp tissues. Interestingly, the overexpression of miR-584 mimics resulted in the inhibition of DPSC proliferation and migration activity; however, it caused a marked reduction in TAZ production. In contrast, the inhibition of miR-584 resulted in the opposite effect. The study noted that miR-584 could be a key regulator of DPSC proliferation, as it affects TAZ expression via the AKT signaling pathway. In addition, they suggested that miR-584 could be considered a biomarker and therapeutic factor for the management of dental diseases [64]. 

It is important to understand the molecular mechanisms underlying the self-renewal ability of DPSCs and their role in the repair and regeneration of the dental pulp following injurious stimuli. A study conducted by Cucco et al. investigated the influence of stem cell factor (SCF) signaling through its receptor tyrosine kinase (c-Kit) on the self-renewal ability of DPSCs. The study performed stable transfection of DPSCs with lentiviral vectors expressing shRNA-c-Kit or vector control. Subsequently, they studied the effect of the SCF/c-Kit axis on DPSC self-renewal ability using a pulpsphere assay under low attachment conditions. Western blotting and flow cytometry were employed to investigate the expression of the polycomb complex protein Bmi-1 (master regulator of self-renewal) [65]. The results of the study revealed the crucial role of the SCF/c-Kit signaling pathway in maintaining pools of stem cells in the DPSCs [65]. 

Long non-coding RNAs (lncRNAs) are found to be expressed in inflamed human dental pulp tissues. The biological functions of lncRNA H19 in DPSCs, including in the differentiation, proliferation, and migration potential of these cells, have been successfully investigated using gain- and loss-of-function experiments with lncRNA H19 and large tumor suppressor 1 (LATS1). A recent study revealed that lncRNA H19 stimulates various biological activities of DPSCs, including differentiation, proliferation, and migration. In contrast, LATS1 inhibits the differentiation, proliferation, and migration of these cells. The authors demonstrated that lncRNA H19 contributes to the differentiation potential of DPSCs because of its suppressive effect on LATS1 by stimulating zeste homolog 2-induced trimethylation of histone 3 at lysine. This could be a promising biomarker and therapeutic target for dental tissue repair and regeneration [66]. Different transfection techniques and transgenes used with DPSCs are summarized in Table 3.

## 6. Discussion

The effective introduction of genetic material into cells to control cell function and behavior is a promising perspective for further use in gene therapy. Both the efficiency of the transfection technique and the quality of the transferred genetic material are important determinants of successful transfection.

Each transfection system has its own distinctive advantages and challenges (Table 4). For example, virus-mediated transfection has several limitations in terms of cost, restricted cargo size, and immunogenicity. Cargo size is not a considerable issue in non-viral transfection systems; however, these systems have lower transfection efficiency. Interestingly, the developed non-viral physical system has demonstrated better efficiency as it is not dependent on the cargo size or transfected cell type.

The efficiency of the non-viral physical transfection system is strongly dependent on the optimization of the parameters utilized during the transfection procedure, which potentially ensures maximum effectiveness and best outcomes. These parameters include voltage, pulse length, amount of plasmid/cargo, and type of buffer used. However, the practical applications of this system may potentially damage the transfected cells, as the use of this system might affect the viability of transfected cells and cause undesirable changes in gene expression. Furthermore, the electric field created in the electroporation transfection system may damage the intracellular organelles and disturb their integrity. 

The development of micro- and nano-technology has improved physical transfection as it only allows for a confined electric field, thereby decreasing damage to the targeted cells and their organelles. Moreover, the combination of multiple transfection approaches aims to enhance transfection efficiency without harming the targeted cells. This hybrid approach combines different non-viral physical systems to improve general transfection outcomes and reduce the shortcomings of each technique. 

DPSC transfection is a promising strategy to enhance the therapeutic benefits of these cells in future regenerative medical and dental approaches. The introduction and control of various stem cell genomes using different transfection genomes can help with sustained delivery and the expression of important molecules [67]. However, the introduction of genes into stem cells has revealed the need to address different issues before moving to the ultimate clinical stage. Challenges related to transfection efficiency, mutagenic potential, and cytotoxicity have been reported. These previous studies should be considered when designing a new delivery system. More meticulous research is needed to improve and optimize the materials and methods for delivering genetic materials into stem cells to improve their therapeutic potential.

## 7. Conclusions

Transfection is the process of delivering genes into cells using various systems. To date, transfection technology has been especially valuable for investigating the roles of different genes in the regenerative potential of various cells, such as DPSCs. In addition, gene delivery has been used to produce proteins, antibodies, and vaccines, and transfection may be used for clinical applications in the future. In this review, we comprehensively discussed various transfection techniques, including viral and non-viral gene delivery systems, which have certain limitations in their applications. In addition, this review sheds light on the availability of transient cell transfections, which can be used to avoid the expensive long-term process of developing stable transfections. We also clarified that no single transfection system can be applied to all cases and all cell types with no side effects. 

This review discusses a variety of available transfection systems applied to investigate the regenerative capacity of dental pulp stem cells, and it encourages further studies on transfection that aim to improve the currently available gene transfection systems. Such studies include the optimization of technique parameters and the stimulation of intracellular delivery with the long-term expression of the genetic material delivered by transfected cells. Moreover, decreasing the toxicity and side effects for human cells should be a focus.

## Figures and Tables

**Table 1:** Key findings of the DPSCs’ properties [21,22,23,24,25,26].

DPSC Properties	Key Findings
Proliferation	DPSCs have high proliferation potential. DPSCs proliferated faster than bone marrow stem cells.
Migration capacity	The migration capacity of stem cells obtained from gingival tissue is higher compared to DPSCs.
Clonogenicity	Colony-forming ability is higher in stem cells obtained from gingival tissue compared to DPSCs.
Angiogenic capacity	Stem cells obtained from gingival tissue showed higher angiogenic capacity compared to cells from DPSCs.
Differentiation potential	The osteogenic differentiation capacity of DPSCs increased along the passages. Stem cells from periodontal tissue revealed increased expression of ALP, calcium deposits, and an early expression of differentiation genes (ALP and COL1A1) compared to DPSCs.

**Table 2:** Descriptive comparison between viral vectors.

Viral Vector	Descriptive Comparisons
Retroviruses	Produces stable transfection Transfects dividing cells Viral genetic materials are enclosed in a capsid and become released upon entering the host cell, and the genome is integrated into the host genome Carries RNAs, which will be reverse transcribed into a double-stranded viral DNA prior to integration with the genome of the host cell for replication and expression Produces an enzyme called “Integrase”, which enhances the integration of the genetic materials into the genome of the host cells Shows low potential in triggering inflammation compared to adenoviruses and herpes viruses; however, it is associated with a high risk of insertional mutagenesis
Adenovirus	Exhibits surface proteins to enhance interaction with the host cell Carries double-stranded DNAs Induces inflammation in the host cell Shows higher packaging capacities than AAVs Has the capacity to transduce most cell types
Adeno-associated virus	Transfects both dividing and non-dividing cells Carries single-stranded DNAs Exhibits safe gene therapy compared to adenoviruses and AAVs as it exerts lower immunogenicity and pathogenicity in humans Delivers large-sized therapeutic genes due to their small packaging capacity (<5 kb)
Herpes virus	Transfects both dividing and non-dividing cells Carries double-stranded DNAs Produce transient transgene expression Has the largest packaging capacity (~150 kb) Delivers specific nucleic acid in treating diseases of the nervous system

**Table 3:** Different transfection techniques and transgenes used with DPSCs.

Transfection Techniques	Goal of the Transfection
Non-viral approach using siRNA to silence the growth/differentiation factor 11 (*GDF11*)	To study the effect of growth/differentiation factor 11 (*GDF11*) on the odontogenic differentiation potential of DPSCs
Viral approach using lentiviral transfection system to introduce the recombinant Wnt4 siRNA into the cells	To study the effect of Wnt4 on the odontogenic and osteogenic differentiation potential of DPSCs
The electroporation-mediated gene delivery approach used to overexpress the recombinant human *GDF11* in the cells	To study the effect of *GDF11* on the healing of injured pulp tissue
Sonoporation is used to transfer the *GDF11* plasmid DNA into the cells	To study the effect of *GDF11* on the odontogenic differentiation potential of DPSCs
Viral approach using lentiviral carriers to deliver VEGF and SDF-1α into the cells	To study the effect of vascular endothelial growth factor (VEGF) on dental pulp regeneration, proliferation, and endothelial cell migration
Viral approach using shRNA to silence the expression of the *VEGFR-2* gene in the targeted cells	To study the effect of vascular endothelial growth factor-2 (*VEGFR-2*) on the dentinogenesis capacity of DPSCs
The electroporation-mediated gene delivery approach used to transfect cells with a transforming growth factor-beta 1 (TGF-B1) gene plasmid	To study the effect of TGF-B1 on the osteogenic and chondrogenic differentiation potential of DPSCs
Non-viral approach using siRNA to overexpress Ena/VASP-like protein (EVL)	To study the effect of EVL on the osteogenic differentiation potential of DPSCs
Non-viral approach using siRNA to silence lysophosphatidic acid (LPA)	To study the effect of LPA on the osteogenic differentiation potential of DPSCs
Viral approach using lentiviral carriers expressing shRNA to modulate the stem cell factor (SCF) signaling through its receptor tyrosine kinase	To study the influence of SCF signaling through its receptor tyrosine kinase on the self-renewal ability of DPSCs

**Table 4:** Advantages and disadvantages of different transfection approaches.

Transfection Technique	Advantages	Disadvantages
siRNA	(1) siRNA has advantages over other small molecular therapeutics as siRNA performs its function through complete base pairing with mRNA (2) Any gene of interest can be targeted by siRNA using the right nucleotide sequence along the targeting mRNA	(1) Naked siRNA might cause inadequate stability and poor pharmacokinetic behavior (2) The phosphodiester bond of siRNA is susceptible to RNases and phosphatases
Electroporation	(1) Optimized electroporation, used alone or in combination with other enhancement methods, expands the range of drugs that can be brought into the cell	(1) Occasionally, it can cause loss of cell homeostasis and/or cell death
Sonoporation	(1) No surgical procedure is required, and it enhances gene transfer with lipofection	(1) Low efficiency of gene transfer (2) Damage to the target cells
Transfection with viral vector	(1) Permanent transfection of non-dividing cells (2) Allows stable, long-term transgene expression (3) High transfection efficiency	(1) Can cause repression or overexpression of transferred genes in the host cell, which might lead to mutagenesis (2) Toxic and immunogenic

## Data Availability

Data sharing is not applicable to this article as no datasets were generated or analyzed during the current study.
